# Lactic Acid Bacteria for Fungal Control and Shelf-Life Extension in Fresh Pasta: Mechanistic Insights and Clean-Label Strategies

**DOI:** 10.3390/molecules31020389

**Published:** 2026-01-22

**Authors:** Noor Sehar, Roberta Pino, Michele Pellegrino, Monica Rosa Loizzo

**Affiliations:** Department of Pharmacy, Health and Nutritional Sciences, University of Calabria, 87036 Rende, CS, Italy; shrnro99a67z236i@studenti.unical.it (N.S.); michele.pellegrino@unical.it (M.P.)

**Keywords:** lactic acid bacteria, antifungal metabolites, *Penicillium*, fresh pasta, bio preservation, clean label, metabolomics, food safety, shelf-life extension

## Abstract

The global food industry is undergoing a major shift driven by increasing consumer demand for clean-label and naturally preserved foods. Fresh pasta is highly vulnerable to fungal damage because of its high water activity (a_w_ > 0.85), typically ranging between 0.92 and 0.97, moderate to near-neutral pH (around 5.0–7.0), and nutrient-rich composition, all of which create favorable conditions for fungal growth during refrigeration, mainly by genera such as *Penicillium* and *Aspergillus*. Fungal contamination results in significant economic losses due to reduced product quality and poses potential health risks associated with mycotoxin production. Although conventional chemical preservatives are relatively effective in preventing spoilage, their use conflicts with clean-label trends and faces growing regulatory and consumer scrutiny. In this context, antifungal lactic acid bacteria (LAB) have emerged as a promising natural alternative for biopreservation. Several LAB strains, particularly those isolated from cereal-based environments (e.g., *Lactobacillus plantarum* and *L. amylovorus*), produce a broad spectrum of antifungal metabolites, including organic acids, phenylalanine-derived acids, cyclic dipeptides, and volatile compounds. These metabolites act synergistically to inhibit fungal growth through multiple mechanisms, such as cytoplasmic acidification, energy depletion, and membrane disruption. However, the application of LAB in fresh pasta production requires overcoming several challenges, including the scale-up from laboratory to industrial processes, the maintenance of metabolic activity within the complex pasta matrix, and the preservation of desirable sensory attributes. Furthermore, regulatory approval (GRAS/QPS status), economic feasibility, and effective consumer communication are crucial for successful commercial implementation. This review analyzes studies published over the past decade on fresh pasta spoilage and the antifungal activity of lactic acid bacteria (LAB), highlighting the progressive refinement of LAB-based biopreservation strategies. The literature demonstrates a transition from early descriptive studies to recent research focused on strain-specific mechanisms and technological integration. Overall, LAB-mediated biopreservation emerges as a sustainable, clean-label approach for extending the shelf life and safety of fresh pasta, with future developments relying on targeted strain selection and synergistic preservation strategies.

## 1. Introduction

The global food industry is undergoing an unprecedented paradigm shift driven by growing consumer awareness of health, sustainability, and food transparency [1]. Within the staple food category, fresh pasta has evolved into a premium product, valued for its superior taste, texture, and artisanal character compared with dried pasta [2]. The market for fresh, refrigerated pasta is experiencing steady growth, fueled by urbanization, increasingly busy lifestyles that favor convenient food solutions, and a rising demand for authentic, high-quality products. Central to this evolution is the strong influence of the so-called clean-label movement.

Modern consumers critically scrutinize ingredient lists in search of foods they perceive as natural, minimally processed, and free from synthetic additives [3]. This trend represents not merely a preference, but a broader desire for simplicity, transparency, and a return to traditional food preservation practices. Consequently, food manufacturers face significant pressure to reformulate and rebrand products by eliminating “chemical-sounding” preservatives such as sorbates and propionates, while still ensuring the levels of safety and shelf life expected by consumers [4]. This challenge is particularly pronounced for highly perishable, high-moisture foods such as fresh pasta, where the removal of conventional preservatives leaves the product especially vulnerable to microbial spoilage.

This vulnerability is intrinsically linked to the physicochemical nature of fresh pasta. Unlike dried pasta, which is characterized by very low water activity (a_w_ < 0.6), fresh pasta typically exhibits high water activity (a_w_ > 0.85) and a near-neutral pH, conditions that strongly favor microbial growth [5]. Its nutrient-rich composition comprising carbohydrates from semolina, along with proteins and lipids, often enhanced by the addition of eggs provides an ideal substrate for spoilage organisms. Among these, filamentous fungi belonging to the genera *Penicillium* and *Aspergillus* are the most frequent and visible contaminants [6]. These molds may originate from raw materials, processing environments, or post-processing handling and lead to rapid quality deterioration. Visible mycelial growth, manifesting as green, blue, or black spots, along with off-flavors and textural degradation, renders the product unsaleable.

More critically, certain fungal species pose potential health risks due to their ability to produce mycotoxins, chemically stable and highly toxic compounds such as ochratoxin A and aflatoxins, which are associated with carcinogenic, immunosuppressive, and other adverse health effects [7]. The significance of fungal spoilage therefore extends beyond economic losses to include serious concerns related to food safety and brand reputation, underscoring the urgent need for effective preservation strategies.

Historically, the fresh pasta industry has relied on a combination of preservation hurdles, including refrigerated storage and modified atmosphere packaging (MAP). However, refrigeration primarily slows microbial growth rather than preventing it, while MAP is costly and not universally effective against all spoilage organisms [8]. Chemical preservatives such as calcium propionate and potassium sorbate have therefore been widely used as direct antifungal agents [9]. Although these compounds are inexpensive and effective, their use conflicts with the clean-label image increasingly demanded by consumers. Growing concerns regarding the potential long-term health effects of chronic exposure have further eroded consumer trust. In addition, repeated use of these preservatives has, in some cases, contributed to the emergence of resistant fungal strains, reducing their efficacy over time [10]. Together, these factors have intensified the demand for natural preservation strategies capable of delivering comparable safety and shelf-life performance without the drawbacks associated with synthetic additives.

Biopreservation has emerged as a scientifically sound and promising solution to this challenge. This approach involves the use of natural or controlled microbiota, or their antimicrobial metabolites, to extend shelf life and enhance food safety [1]. Among biological agents, lactic acid bacteria (LAB) are particularly well suited for the biopreservation of cereal-based products such as fresh pasta [11]. LAB have a long history of safe use in fermented foods such as yogurt, cheese, and sourdough bread; consequently, many species have been granted Generally Recognized as Safe (GRAS) or Qualified Presumption of Safety (QPS) status [12].

The antifungal activity of LAB is not based on a single mechanism but rather based on a multifaceted and synergistic arsenal. LAB primarily produce organic acids, such as lactic and acetic acids, which lower pH and create unfavorable conditions for fungal growth [13]. In addition, many antifungal LAB strains synthesize low-molecular-weight metabolites, including phenyl lactic acid, cyclic dipeptides, and volatile compounds such as diacetyl. Acting in combination, these compounds can disrupt fungal cell membranes, interfere with energy metabolism, and induce morphological damage, thereby providing a robust and natural antifungal barrier. Importantly, LAB-based preservation aligns well with clean-label requirements, as ingredients may be declared simply to be “lactic acid bacteria” or “fermented flour” [14].

Despite their considerable potential, the translation of antifungal LAB from laboratory-scale research to industrial fresh pasta production requires a comprehensive and critical assessment of existing knowledge. While numerous reviews have addressed antifungal LAB applications, most focus on dairy, meat, or sourdough systems, whereas the specific challenges and opportunities associated with fresh pasta remain underexplored.

This review offers a critical synthesis of research from the past decade on fresh pasta spoilage, the microbial dynamics that drive it, and the gradual advancement of lactic acid bacteria (LAB)-based antifungal biopreservation strategies. Initial studies during this period mainly clarified the inhibitory role of LAB-derived metabolites against fungal growth. In contrast, more recent work has shifted toward strain-specific effects, detailed elucidation of modes of action, and the technological integration of LAB into fresh pasta systems. By consolidating these developments, this review shows that LAB-mediated biopreservation has progressed into a robust, sustainable, and clean-label-compatible strategy for enhancing the shelf life and safety of fresh pasta, supported by growing experimental evidence. Looking ahead, further progress is expected to depend on targeted strain selection, synergistic combinations with complementary preservation hurdles, and application approaches adapted to product formulation and processing conditions, thereby extending the relevance of this strategy to other high-moisture, cereal-based foods.

**Figure 1:**
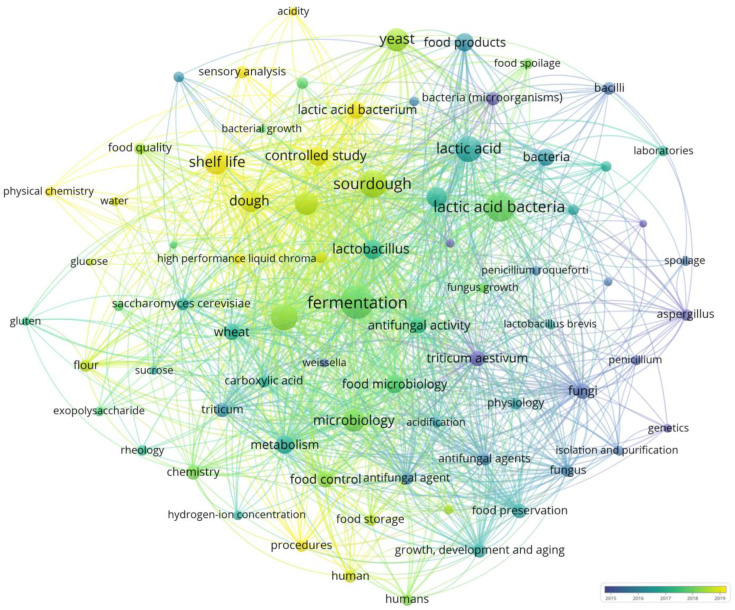
Bibliometric network map of keywords related to the use of lactic acid bacteria (LAB) for fungal control and shelf-life extension in fresh pasta. Nodes represent keywords extracted from the scientific literature, while links indicate co-occurrence relationships between terms. Node size is proportional to keyword frequency. Node colors reflect the temporal evolution of publications, ranging from blue (earlier years) to yellow (more recent years).

Accordingly, this review aims to summarize and critically evaluate the literature of the last ten years to help define future directions (Figure 1).

This review uses a formal analytical framework to critically evaluate studies on antifungal lactic acid bacteria (LAB), treating them as equivalent. The framework considers four important attributes, including (i) ecological relevance of strain source, (ii) intensity and particularity of antifungal actions, (iii) degree of validation (in vitro or in situ pasta systems), and (iv) technological implications in industrial production of fresh pasta. Ultimately, this review intends to serve as a comprehensive resource supporting the development of safer, longer-lasting, and genuinely clean-label fresh pasta products. In contrast to earlier reviews that have concentrated on LAB antifungal metabolites, in general, this review has included mechanistic information as well as application outcomes of these agents in cereal-based systems, but with a special gentle focus on fresh pasta and its preservation limitations.

## 2. The Spoilage Challenge in Fresh Pasta

Fresh pasta represents a significant segment of the growing market for premium and convenient food products; however, it is highly susceptible to spoilage due to its inherent physicochemical characteristics [15]. Unlike dried pasta, fresh pasta has a high moisture content, which strongly supports microbial growth. Spoilage in fresh pasta is a multifactorial issue that extends beyond intrinsic product properties and specific microbial contaminants, encompassing substantial economic losses and serious food safety concerns [11]. Understanding these challenges is essential for the development of effective preservation strategies that not only maintain product quality but also enhance consumer safety. The interaction between the physical and chemical characteristics of fresh pasta and the surrounding preservation environment is highly complex, requiring comprehensive knowledge and the implementation of targeted intervention strategies.

### 2.1. Intrinsic Factors Favoring Microbial Growth

#### 2.1.1. High Water Activity and Nutrient Composition

The main weakness of fresh pasta is its high water activity (a_w_ > 0.85), typically ranging between 0.92 and 0.97 [16]. This is a favorable microbial response because water availability permits all the necessary biological functions such as transport of nutrients, enzyme action, and cellular reaction. Unlike dry pasta, where microbiological stability is ensured by low water activity (a_w_ less than 0.60) [17], fresh pasta is provided with enough free water to allow microorganisms, yeast, and molds to grow. It has a high water activity and therefore is conducive to the growth of filamentous fungi, particularly Penicillium and *Aspergillus* species, dominating spoilage in fresh pasta, as summarized in Table 1 [18]. Although fungal growth thrives optimally under such conditions, bacterial growth is more severely limited by pH, low storage temperature, and microbial competition in the fresh pasta ecosystem [11]. Consequently, although the levels of water activity are permissible, bacteria normally contribute to spoilage to a secondary extent, and fungal growth is the most important contributor to fresh pasta deterioration during refrigerated shelf life [17]. The importance of water activity cannot be overestimated, as it is the main factor in microbial vulnerability in food products, and the typical texture and quality of fresh pasta are inevitably connected to its high water content [18]. Making the issue of water activity even harder, the product has a high nutritional value. The carbohydrate matrix is mainly made of semolina or durum wheat flour and offers a vast amount of glucose, maltose, and other small sugars that act as short-term sources of energy to the microorganisms [19]. In addition, gluten proteins also provide a sustained nitrogen supply that helps to support microbial growth with proteolytic activity [20]. Such a nutritional load is made all the heavier in egg-enriched formulations. The eggs provide quality protein, lipids, vitamins, and minerals, which convert the pasta to a complete microbial growth medium [21]. The lipid component, which is highly prone to enzymatic degradation, may contribute to secondary spoilage expressions such as rancidity and off-flavors, concomitantly contributing other sources of carbon to microbial metabolism [22].

#### 2.1.2. The Role of pH and Formulation (Egg-Based vs. Egg-Free)

The pH profile of fresh pasta further contributes to its susceptibility to spoilage. Most fresh pasta products exhibit a pH ranging between 5.0 and 7.0, a range that coincides with the optimal growth conditions for many spoilage microorganisms [2]. Unlike acidic foods, such as fermented products, which benefit from an inherent inhibitory hurdle, fresh pasta lacks a natural acid-based defense mechanism, leaving it particularly exposed to microbial proliferation.

Product formulation plays a critical role in shaping microbial selection pressure and spoilage dynamics. In particular, the increased nutritional complexity of egg-enriched pasta promotes the development of more diverse microbial communities capable of metabolizing a broader array of protein and lipid substrates [23]. As a result, egg-based pasta often exhibits faster spoilage rates and may support microbial populations distinct from those found in egg-free formulations. Formulation-dependent effects extend beyond nutrient composition alone. Egg-derived proteins and lipids can interact with water molecules, influencing both the distribution and availability of water within the pasta matrix. In addition, even minor variations in pH among recipes or brands may preferentially favor certain microbial groups over others. For example, specific *Penicillium* species have different pH optima compared with *Aspergillus* species. Thus, subtle formulation differences may predetermine the dominant spoilage organism in each product [24].

The absence of strong intrinsic preservation hurdles implies that the microbial stability of fresh pasta must rely largely on extrinsic control measures and deliberately applied preservation strategies.

### 2.2. Primary Fungal Spoilers and Contamination Routes

#### 2.2.1. Dominant Genera: Penicillium and Aspergillus

Filamentous fungi belonging to the mycobiota dominate the spoilage of fresh pasta, with each genus presenting distinct and significant implications for product quality and safety. Among these, *Penicillium* species represent the most prevalent spoilage agents. Frequently isolated species include *P. rubens* (formerly *P. chrysogenum*), followed by *P. roqueforti* and *P. expansum*, all of which are commonly associated with fresh pasta deterioration [11,25]. Their psychotropic nature, enabling growth at refrigeration temperatures, combined with rapid colonization rates, makes these species particularly problematic in chilled products [26]. Spoilage by *Penicillium* is typically characterized by the formation of blue-green sporulating colonies, making contamination readily visible to consumers. Another important group of spoilage organisms is represented by *Aspergillus* species, notably *A. flavus* and *A. niger*. These fungi are of particular concern not only because of their spoilage potential but also due to their capacity to produce mycotoxins [27]. Although *Aspergillus* species generally exhibit higher optimal growth temperatures than *Penicillium*, several strains are nevertheless capable of growing under refrigeration conditions, contributing to spoilage during cold storage [28].

The genus *Aspergillus*, comprising xerophilic species such as *A. repens* (formerly *Eurotium repens*) and *A. ruber* (formerly *E. rubrum*), represents a notable exception within the fresh pasta spoilage ecosystem. Despite the high water activity of fresh pasta, *Aspergillus* species may outcompete other fungi under conditions of localized surface drying or when introduced via heavily contaminated raw materials [29]. Their ability to grow at relatively low water activities and to produce stress-resistant spores makes them particularly persistent in production environments. Table 1 summarizes the principal fungal spoilage organisms associated with fresh pasta, highlighting their key characteristics and the risks they pose to product quality and safety.

**Table 1:** Primary fungal spoilers in fresh pasta.

Fungal Genus	Common Appearance	Optimal Growth Condition	Primary Risk	Key Mycotoxins of Concern	Ref.
*Penicillium* spp.	Blue-green spore masses, rapid surface colonization	Psychrotrophic (grows at refrigeration temps: 4–8 °C), high a_w_ (>0.85), pH 5–7	Visual spoilage, off-flavors, texture degradation	Ochratoxin A (*P. verrucosum*), Patulin (*P. expansum*)	[11,25]
*Aspergillus flavus*, *A. niger*	Yellow green to black powdery growth	Mesophilic (25–35 °C) but can grow at 8–10 °C, a_w_ > 0.80, pH 3–7	Mycotoxin production, visible spoilage	Aflatoxins (B1, B2, G1, G2)—potent hepatocarcinogens	[27]
*Aspergillus* spp. (other)	Diverse colors: yellow, brown, black	Moderate a_w_ (0.75–0.85), pH 3–7	Mycotoxin risk, discoloration	Ochratoxin A (*A. ochraceus*)	[28]
*Eurotium repens*, *E. rubrum*	Yellow to orange cleistothecia, often with surface drying	Xerophilic (low a_w_: 0.70–0.75), pH 4–7	Persistent contamination from raw materials, surface spoilage	Non-toxigenic, but indicators of poor storage	[29]
Other contaminants (e.g., *Cladosporium*, *Rhizopus*)	Dark green/black spots, fuzzy growth	Variable, often high humidity	Visual defects, odor issues	Rarely toxigenic in pasta systems	

#### 2.2.2. Production Chain Sources

Spoilage fungi can be introduced into fresh pasta through multiple and widespread contamination routes within the production ecosystem. Raw materials, particularly wheat flour, represent the primary source of contamination and typically contain fungal spore loads ranging from 10^2^ to 10^4^ spores per gram [30]. These spores are highly resistant to conventional milling processes and are therefore readily incorporated into the pasta dough. As a result, flour constitutes a substantial inoculum reservoir that must be carefully managed through raw material selection and, where appropriate, additional processing interventions [30,31].

The processing environment itself is another major source of continual contamination. Airborne fungal spores may deposit on product surfaces during critical processing steps such as mixing, sheeting, cutting, and packaging [32]. Environmental monitoring studies conducted in pasta manufacturing facilities have demonstrated a direct relationship between air quality and product spoilage rates. In addition, equipment surfaces represent significant contamination reservoirs. Complex machinery, including extruders, cutters, and conveyor systems, provides numerous niches for biofilm formation and spore accumulation, thereby facilitating persistent contamination [33].

The packaging stage introduces additional risks, as packaging materials stored under non-sterile conditions may harbor fungal spores that can come into direct contact with the product surface. Moreover, human activity within the production facility contributes to cross-contamination through handling and movement between processing areas, further complicating control efforts. Consequently, comprehensive environmental monitoring, rigorous sanitation protocols, and effective hygiene management programs are essential components of quality assurance systems aimed at minimizing fungal spoilage in fresh pasta production [34,35].

### 2.3. Spoilage Effects: Risk of Quality and Mycotoxins

Fungal spoilage in fresh pasta extends beyond the direct deterioration of product quality and may pose significant risks to human health [7,36]. The most immediately apparent quality defect is the visible development of mycelial growth, characterized by colored sporulation, commonly green, blue, black, or white, that renders the product unacceptable to consumers [37]. This visual contamination is often accompanied by the production of microbial volatile organic compounds (MVOCs), which generate characteristic musty, earthy, or mushroom-like odors. Compounds such as geosmin and 1-octen-3-ol can be detected at extremely low concentrations and may negatively affect sensory perception even before visible fungal growth becomes apparent [38].

Beyond sensory defects, spoilage fungi cause progressive textural degradation through the activity of extracellular enzymes, including proteases, amylases, and lipases [39]. Proteolytic enzymes disrupt the gluten network, leading to softening, loss of elasticity, and ultimately slimy or mushy textures. Amylolytic activity results in starch breakdown, causing surface stickiness and, in some cases, adhesion of the product to packaging materials [40]. In egg-based pasta, lipolytic enzymes further contribute to spoilage by degrading fats, promoting rancid off-flavors, and diminishing nutritional value. These biochemical and physical changes act synergistically, accelerating quality deterioration and significantly shortening the shelf life of fresh pasta. Beyond quality losses, the most serious consequence of fungal spoilage is the potential production of mycotoxins [41]. Several *Penicillium* and *Aspergillus* species produce toxic secondary metabolites associated with severe health hazards. Ochratoxin A, produced by *P. verrucosum* and certain *Aspergillus* species, is nephrotoxic, immunotoxic, and potentially carcinogenic [42]. Aflatoxins, predominantly synthesized by *Aspergillus flavus* and *A. parasiticus*, rank among the most potent hepatocarcinogens identified to date. A critical concern associated with mycotoxins is their chemical stability: they may persist even after visible mold growth has been removed and can survive typical cooking processes, posing an unseen risk that cannot be mitigated by trimming contaminated areas alone [43].

Given that mycotoxin levels in foods are strictly regulated worldwide, non-compliance can result in product recalls, legal action, and irreparable damage to brand reputation. Consequently, the control of toxigenic fungi in fresh pasta is not merely a quality issue but a fundamental food safety requirement that demands robust and preventive management strategies [11].

## 3. Antifungal Lactic Acid Bacteria: Sources and Selection

The selection of suitable lactic acid bacteria (LAB) strains is a critical determinant in the successful establishment of an effective biopreservation strategy for fresh pasta [44]. This process extends far beyond the mere identification of antifungal-active strains under laboratory conditions. Instead, it requires a systematic and structured approach that prioritizes the isolation of candidate strains from relevant ecological niches, relies on robust screening methodologies capable of predicting real-world performance, and applies stringent selection criteria to identify strains that not only exhibit strong antifungal activity but are also compatible with fresh pasta production. The complex, multicomponent structure of fresh pasta, together with the technological constraints of industrial processing and the need to meet consumer expectations, necessitates a multistep strain selection framework [11]. Achieving success in this context requires a thorough understanding of the ecological interactions between LAB and their native environments, particularly cereal-based ecosystems, where these microorganisms have coevolved alongside the predominant fungal spoilage species. The selection of LAB in fresh pasta systems is also limited by the requirement of preserving product texture, color, and sensory neutrality during refrigerated storage as opposed to sourdough or baked cereal.

### 3.1. Promising LAB Species from Cereal Environments

The antifungal lactic acid bacteria used in the processing of fresh pasta are selected based on functional and technological properties as opposed to taxonomy [31,32]. The operationally defined idea in this review is that cereal-adapted LAB refers to strains that have been initially isolated from cereal-based systems that experience stable growth, metabolic activity, and antifungal activity in carbohydrate-based cereal matrices. The selection of these strains via natural selection occurs in the grain ecosystems as they gain competitive fitness, the ability to utilize substrates efficiently, and antifungal strategies, which enable them to overcome populations of fungi which can be associated with the cereals [33,34,37]. In that case, the strain selection is biased toward ecological origin and technological stability in refrigerated storage, as well as capacity to generate antifungal metabolites without adversely affecting product quality [39]. Ecosystems that are based on cereals are thus the most logical and fruitful in terms of isolating LAB that can be applied in fresh pasta biopreservation [12,45]. Among the most extensively studied and promising species is *Lactiplantibacillus plantarum* (formerly *Lactobacillus plantarum*), a highly versatile and adaptable bacterium widely distributed across fermented foods and plant-associated environments [46]. Its prevalence in sourdough ecosystems is particularly relevant, as these systems share several physicochemical and nutritional characteristics with fresh pasta matrices. Numerous *L. plantarum* strains have been shown to produce a broad spectrum of antifungal metabolites, including phenolic acids, notably phenyllactic acid (PLA), cyclic dipeptides, hydroxy fatty acids, and various organic acids (Table 2) [47]. The metabolic flexibility of *L. plantarum* allows it to utilize diverse carbon sources present in wheat flour, enabling sustained growth and metabolic activity within pasta matrices. Furthermore, many strains possess multiple genetically encoded antifungal mechanisms, conferring broad-spectrum activity and reducing the likelihood of resistance development in target fungi [48].

*Ligilactobacillus amylovorus* (formerly *Lactobacillus amylovorus*) is another highly promising candidate, particularly due to its strong ecological association with cereal-based habitats. Originally isolated from plant materials and sourdough, this species has demonstrated remarkable adaptation to grain-derived substrates [49]. Notably, *L. amylovorus* produces potent antifungal compounds when cultured in cereal-based media, suggesting that its metabolic pathways are especially optimized for cereal environments. Strains such as *L. amylovorus* DSM 19280 have shown exceptional antifungal efficacy in cereal-based systems, including sourdough bread and malted barley, indicating their capacity to express antifungal activity in complex food matrices such as fresh pasta [50]. The antifungal activity of *Ligilactobacillus amylovorus* is often attributed to synergistic mixtures of organic acids and other bioactive metabolites that are effective against common pasta spoilage fungi, particularly *Penicillium* species. Other LAB species, although less extensively studied, have also demonstrated significant potential. *Levilactobacillus brevis* (formerly *Lactobacillus brevis*), a microorganism commonly associated with sourdough and fermented grains, has been reported to produce effective antifungal metabolite profiles, particularly when cultivated in wheat-based media [51,52,53]. Several *L. brevis* strains synthesize cyclic dipeptides with documented antifungal activity. *Limosilactobacillus reuteri* (formerly *Lactobacillus reuteri*) represents a distinct case due to its ability to produce reuterin, a broad-spectrum antimicrobial compound generated in the presence of glycerol [54]. *L. reuteri* gives rise to reuterin with the presence of glycerol; the antifungal activity of the product in fresh pasta is restricted by the naturally low glycerol concentration level of the matrix. While fresh pasta typically contains low glycerol levels, this example highlights the metabolic versatility of LAB and suggests that formulation or process optimization could be employed to enhance antifungal efficacy.

In addition to these well-characterized species, emerging research is exploring the potential of less conventional LAB originating from cereal fermentations. For instance, *Lentilactobacillus harbinensis* (formerly *Lactobacillus harbinensis*), isolated from traditional Chinese fermented cereals, has exhibited antifungal activity in dairy systems and may hold promise for application in pasta matrices [55]. Similarly, several *Pediococcus* species, particularly *P. pentosaceus*, have demonstrated antifungal properties in cereal-based studies [56].

The ecological rationale for sourcing LAB from cereal environments is well founded. These strains have coevolved alongside the fungi that commonly contaminate cereal grains and cereal-derived products, resulting in competitive strategies that often translate into high-affinity and effective antifungal activity. Such evolutionary adaptation is frequently associated with metabolic traits advantageous for fresh pasta applications, including amylolytic activity that facilitates starch utilization and supports microbial persistence throughout product shelf life [57,58]. Moreover, cereal-adapted LAB are typically well suited to the water activity and pH conditions characteristic of fresh pasta storage. Notably, many of these strains can inhibit the most problematic fungal genera associated with pasta spoilage, including *Penicillium* and *Aspergillus*, thereby allowing their coevolutionary relationships to be strategically exploited for biopreservation purposes [59].

Although *L. plantarum* is the most frequently reported antifungal species, its prevalence in the literature can be partly attributed to a research bias toward sourdough systems rather than to its actual effectiveness in fresh pasta. In contrast, *L. amylovorus* has been scarcely investigated in pasta matrices, despite its superior adaptation to cereal substrates and its high production of antifungal metabolites during low-temperature storage. Table 2 presents promising antifungal LAB strains that have been reported in the literature to be used in cereal-based applications and also their natural sources, key metabolites, and the type of antifungal inhibition. It is important to note that most of the studies give qualitative or mechanistic antifungal evidence gained under in vitro or model-system conditions. Quantitative efficacy values and in situ validation in fresh pasta systems are also scarcely known and hence warrant direct comparison of strains against each other and highlight the necessity to standardize pasta-based challenge tests.

**Table 2:** Promising antifungal LAB strains for cereal-based applications.

Lab Strains	Natural Habitat/Sources	Key Antifungal Metabolites	Reported Efficacy Against	Type of Antifungal Evidence	Ref.
*Lactiplantibacillus plantarum*	Sourdough, fermented cereals, plant material	PLA, cyclic dipeptides (e.g., cyclo(Phe-Pro), cyclo(Leu-Pro)), organic acids, hydroxy fatty acids	*Penicillium* spp., *Aspergillus* spp.	In vitro growth inhibition assays	[48]
*Ligilactobacillus amylovorus*	Cereal grains, sourdough, malted barley	Organic acids, PLA, antifungal peptides	*Penicillium chrysogenum*, *A. flavus*	Agar diffusion and metabolite identification	[49]
*Levilactobacillus brevis*	Sourdough, fermented grains	Cyclic dipeptides (e.g., cyclo(Phe-Pro), cyclo(Leu-Pro)), organic acids, PLA (strain-dependent)	*Penicillium* spp., *Fusarium* spp.	Dual-culture inhibition assays	[53]
*Limosilactobacillus reuteri*	Human/animal gut, some fermented foods	Reuterin (from glycerol), organic acids	Broad spectrum (fungi and bacteria)	In vitro inhibition (glycerol-supplemented media)	[54]
*Lentilactobacillus harbinensis*	Traditional Chinese fermented cereals	PLA, acetic acid, other phenolic acids	*Penicillium*, *Aspergillus* (in dairy models)	In vitro inhibition	[55]
*Pediococcus pentosaceus*	Fermented cereals, vegetables, meats	Organic acids, bacteriocins, cyclic peptides (strain-dependent)	*Penicillium*, *Aspergillus* (in dairy models)	In vitro inhibition	[56]

### 3.2. Strategies for Screening and Validating Antifungal Activities

The development of an effective screening strategy for antifungal lactic acid bacteria requires a multistage approach, beginning with high-throughput screening and progressing toward increasingly complex and application-relevant validation phases. The initial screening step typically relies on agar-based assays, which allow the rapid evaluation of large strain collections [60]. Among these, the dual-culture assay is one of the most used techniques, in which LAB and target fungi are inoculated on opposite sides of an agar plate and antifungal activity is assessed based on the resulting inhibition zone. While this method provides valuable preliminary information, it has notable limitations, particularly its inability to distinguish among different inhibition mechanisms [61].

Additional agar-based methods, such as the agar spot test and well-diffusion assays using cell-free supernatants (CFSs), offer further insights. The latter are especially useful for determining whether antifungal activity is mediated by diffusible metabolites rather than solely by nutrient competition or pH reduction [62]. A critical consideration at this stage is the choice of growth medium. Conventional laboratory media, such as de Man–Rogosa–Sharpe (MRS) broth, are suitable for LAB cultivation but often contain components that may mask or artificially enhance antifungal activity [63]. In particular, the presence of acetate in standard MRS formulations can confound results by contributing antifungal effects that are not representative of those occurring in fresh pasta systems. Consequently, modified MRS media lacking acetate, or preferably cereal-based media such as wheat flour hydrolysates or pasta-simulating substrates, provide more predictive screening outcomes [64]. These media more accurately reflect the real food matrix and influence LAB metabolic pathways, potentially leading to the production of antifungal metabolites distinct from those synthesized in nutrient-rich laboratory media.

Following primary screening, promising candidates are subjected to more advanced quantitative evaluations in liquid systems. This stage typically involves culturing LAB in appropriate media and assessing the antifungal activity of their CFS using microtiter plate-based assays. Such approaches enable the determination of minimum inhibitory concentrations (MICs) and facilitate the study of fungal inhibition dynamics [65]. At this stage, broad-spectrum antifungal activity is considered essential, and assessments should be conducted using panels of relevant spoilage fungi, including multiple strains of *Penicillium* and *Aspergillus*, given that sensitivity to antifungal metabolites may vary markedly among species and strains.

The most critical transition in the screening process is the shift from in vitro assays to pasta-relevant validation systems. This progression typically begins with simplified model systems, such as pasta-like agar or dough-based matrices, which help identify strains capable of expressing antifungal activity in solid or semi-solid substrates. Ultimately, candidate LAB must be evaluated in challenge tests using real fresh pasta. These tests involve incorporating selected LAB strains into pasta formulations, deliberately inoculating the product with target spoilage fungi, and monitoring fungal growth, metabolic activity, and potential mycotoxin production during storage under realistic conditions [66].

Recent screening strategies increasingly incorporate molecular and analytical approaches to enhance efficiency and mechanistic understanding. Genetic screening for known antifungal-related genes can provide early indications of a strain’s potential, although such predictions must be confirmed through phenotypic assays [67]. Metabolomic analyses using techniques such as Liquid Chromatography–Mass Spectrometry (LC–MS) or Gas Chromatography–Mass Spectrometry (GC–MS) allow detailed characterization of the antifungal compounds produced by promising strains, offering insights into their modes of action and enabling the identification of strains that generate novel or synergistic metabolite profiles [68]. This mechanistic knowledge can support not only strain selection but also process optimization aimed at maximizing the production of key antifungal metabolites.

Finally, the validation process should include an assessment of how processing conditions influence LAB viability and activity. Factors such as mixing time, extrusion pressure, and, where applicable, drying or cooling conditions may significantly affect the performance of bioprotective cultures [69]. Early evaluation of these interactions allows for the selection of LAB strains that are not only strongly antifungal but also sufficiently robust to withstand industrial manufacturing stresses.

### 3.3. Criteria in the Selection of Strains Used in Pasta

Although antifungal activity represents the primary selection criterion, a range of additional factors must be considered when identifying lactic acid bacteria strains suitable for application in fresh pasta biopreservation. These criteria are essential to ensure that selected strains are not only effective but also technologically feasible, safe, and acceptable for commercial production.

A key aspect of technological suitability is the ability of a strain to survive, persist, and remain metabolically active within the fresh pasta matrix throughout the intended shelf life. This requires tolerance to the intrinsic conditions of fresh pasta, including water activity, pH, and redox potential, as well as the capacity to compete with the indigenous microbiota associated with raw materials [11,70]. Closely following antifungal efficacy, the impact of the selected strains on product quality is of paramount importance.

Ideally, bioprotective LAB strains should be organoleptically neutral, meaning that they do not induce undesirable changes in flavor, aroma, or texture [71]. Certain LAB strains are known to produce diacetyl, resulting in buttery notes that may be inappropriate for fresh pasta, while others can generate excessive acidification, altering the product’s sensory profile [72]. In addition, pronounced proteolytic or lipolytic activity may lead to texture softening or the development of rancid off-flavors, particularly in egg-enriched formulations [73]. Consequently, rigorous sensory evaluation using difference testing and descriptive analysis with trained panels is essential to confirm that the inclusion of a bioprotective culture does not compromise product quality.

Safety represents another non-negotiable selection criterion. Although LAB are generally regarded as safe, strain-specific assessments remain necessary. These evaluations include molecular confirmation of species identity, screening for antibiotic resistance determinants (especially acquired resistance genes), and assessment of the potential for biogenic amine production [74]. Strains intended for food applications should ideally possess Generally Recognized as Safe (GRAS) status or be eligible for Qualified Presumption of Safety (QPS) designation, both of which require comprehensive documentation of strain safety [75]. For strains lacking established regulatory status, an even more thorough safety assessment is mandatory.

Practical considerations related to industrial production also strongly influence strain selection. Suitable LAB strains must be amenable to large-scale, cost-effective fermentation, and their stability during concentration, drying (if applicable), and storage is a key determinant of commercial viability [76]. High survival and retained activity following preservation processes such as freeze-drying or spray-drying are particularly advantageous for industrial applications [77]. Moreover, strains that can be readily integrated into existing production workflows offer a clear operational advantage.

Regulatory and labeling implications must also be carefully evaluated. In many markets, LAB used for biopreservation can be declared as cultures or fermented ingredients rather than as preservatives, aligning well with clean-label expectations [3]. However, familiarity with the regulatory framework of target markets remains essential, as it may ultimately determine the feasibility of commercial adoption.

Finally, the economic viability of a given strain must be assessed. This encompasses not only the cost of culture production but also the effective dosage required for antifungal protection, the potential to reduce reliance on other preservation hurdles (such as modified atmosphere packaging or chemical preservatives), and the economic benefits associated with extended shelf life, including reduced food waste and expanded distribution opportunities [78]. Strains that achieve effective protection at lower inoculation levels are particularly attractive, as they combine enhanced performance with improved cost efficiency.

## 4. Mechanisms of Action and Antifungal Metabolites of Primary Interest

**Figure 2:**
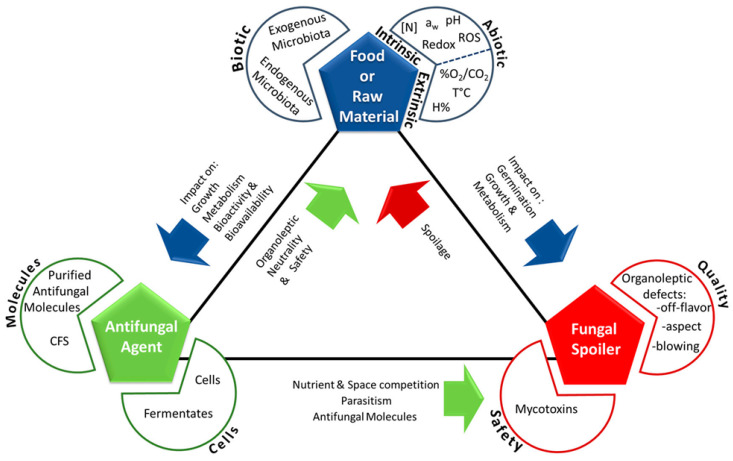
Food biopreservation systems. Interactions that occur between fungal spoilers, antifungal microbial agents, and the food matrix. Leyva Salas et al. [80].

The antifungal activity of lactic acid bacteria in fresh pasta is a multifaceted biological phenomenon involving numerous mechanisms and a wide array of antimicrobial metabolites. Unlike single-molecule chemical preservatives that target specific cellular processes, antifungal LAB employ a complex, multicomponent strategy in which several metabolites act synergistically to inhibit fungal growth. This “combination therapy” effect, delivered through a single biological system, offers several advantages, including a reduced likelihood of resistance development, broader antifungal spectra, and enhanced overall efficacy through synergistic interactions among metabolites [79]. These antifungal mechanisms should be considered in the context of fresh pasta, which is characterized by high water activity, a short shelf life, and the absence of a thermal kill step, factors that may influence the efficacy of antifungal metabolites compared with baked cereal products. These antifungal mechanisms operate at both chemical and cellular levels and are particularly well suited to the fresh pasta matrix, where multiple intrinsic and extrinsic factors influence spoilage dynamics. A thorough understanding of these mechanisms is essential for selecting LAB strains with optimal antifungal performance and for designing effective strategies to control fungal spoilage. As illustrated in Figure 2, the interactions among food components, spoilage fungi, and antifungal agents are multidirectional and highly complex, underscoring the need for an integrated approach to biopreservation in fresh pasta systems.

### 4.1. The Antifungal Metabolite Cocktail

#### 4.1.1. Organic Acids: Lactic and Acetic Acid

The main antifungal metabolites found in fresh pasta are organic acids, primarily lactic and acetic acid, which are generated by LAB in fresh pasta systems [81,82]. They act through their antifungal activity, mostly linked to intracellular acidification due to diffusion of the undissociated acid form into fungal cells, leading to energy depletion and the disruption of metabolism [83,84]. Although lactic acid also plays a major role in decreasing the pH, acetic acid has much stronger antifungal potential, as it has a higher percentage of undissociated molecules at pasta pH and directly inhibits key metabolic enzymes [65,85]. Notably, the two acids are synergistic and increase membrane permeability and intracellular stress, and all of this intensifies the antifungal activity of fresh pasta matrices.

#### 4.1.2. Phenyllactic Acid and Other Phenolic Compounds

Phenyllactic acid (PLA) is one of the most applicable LAB-derived antifungal metabolites, with its stability, broad spectrum, and potent effect on anti-germination [86,87]. PLA interferes with the membrane and cell wall structure of fungi, causing morphological defects and growth retardation. Compounds that are structurally related to 4-hydroxyphenyllactic acid and indole-3-lactic acid, in turn, also increase the antifungal effect by synergizing with PLA [88]. Besides inhibition of growth, PLA has also been observed to suppress mycotoxin biosynthesis at levels that are considered sublethal, thus giving PLA an essential safety benefit to cereal-based foods [89]. The production of these metabolites is very strain- and matrix-sensitive [46].

#### 4.1.3. Volatile Compounds and Cyclic Dipeptides

Cyclic dipeptides (such as cyclo(Phe-Pro) and cyclo(Leu-Pro)) are involved in the LAB antifungal effect by membrane destabilization and membrane gradient disruption [11]. They have a cyclic and amphiphilic structure that improves stability and cellular interaction. Simultaneously, volatile organic compounds produced by LAB cause fungal growth inhibition by protein modification and metabolic interference and are applicable to packaged fresh pasta systems [90,91]. These compounds frequently exhibit moderate single actions, but when they coexist, a high level of synergistic antifungal activity is realized.

These compounds were shown to be antifungal in the laboratory and in simple food systems, but their performance in fresh pasta food has not yet been systematically tested. Most studies that are available report inhibition in laboratory conditions or in non-pasta cereal products, and quantitative preservation performance in fresh pasta has not been reported extensively. Thus, the existing data has justified the possible usage of these compounds as supplementary antifungal agents, but in situ pasta-based research must be performed to prove their practical applicability.

### 4.2. Synergistic Effects and Modes of Action

Metabolites produced by LAB have synergistic effects in interfering with fungal energy metabolism, membrane stability, and cellular homeostasis. Intracellular acidification and ATP depletion are caused by weak acids, and cyclic dipeptides and VOCs disrupt mitochondrial respiration and oxidative phosphorylation [92,93]. At the same time, hydrophobic metabolites destabilize the plasma membranes and weaken the cell wall, allowing intracellular components to leak through and causing damage to the cell irreparably [94,95]. The integrated processes can describe the extensive and strong antifungal effect of the LAB-based biopreservation systems. Although there is a substantial body of mechanistic evidence, comparatively very few studies have tested the actual preservation performance of antifungal LAB in actual food systems. According to the available data, there are strong factors in antifungal effectiveness that are determined by the selection of strain, food matrix formulation, the temperature of storage, and the mode of application. LAB have been found to slow the appearance of visible mold in cereal products, but quantitative shelf-life extension information and controlled but otherwise in situ challenge studies are limited, especially as regards fresh pasta. This gap reflects the necessity to apply research to supplement mechanistic knowledge.

### 4.3. Effect of Pasta Matrix on Metabolite Production and Effectiveness

The compositional and structural characteristics of fresh pasta exert a profound influence on the antifungal efficacy of LAB, as the food matrix is not a passive background but rather an active determinant of microbial behavior.

Nutrient availability directly modulates LAB metabolism: carbohydrates derived from wheat stimulate the production of organic acids, while amino acids such as phenylalanine and tyrosine serve as key precursors for PLA and other phenolic metabolites [96]. In egg-based pasta, the presence of additional proteins and lipids may further alter metabolic pathways, enhance LAB biomass, or promote the synthesis of fatty acid-derived antifungal compounds [97].

Physical properties of the pasta matrix also play a critical role. The dense gluten network can limit the diffusion of larger molecules while favoring the migration of smaller or more hydrophobic metabolites, including organic acids and PLA. Surface characteristics such as microporosity and the presence of lipid films further influence the localization, diffusion routes, and sites of action of antifungal compounds on contaminating fungi [11].

Intrinsic parameters such as water activity (a_w_) and pH also modulate LAB performance. The high water activity and moderately acidic pH (5.5–6.5) characteristic of fresh pasta support LAB growth while increasing the proportion of undissociated weak acids, thereby enhancing antimicrobial activity. This effect is further amplified as LAB fermentation progressively lowers the pH during storage [18].

Conversely, interactions between pasta components and LAB metabolites may reduce immediate antifungal efficacy. Proteins can bind phenolic compounds, while starch matrices may entrap organic acids, delaying their availability. However, such interactions may also create slow-release reservoirs of antifungal metabolites that contribute to prolonged protection during storage. Owing to these complex matrix effects, LAB strains that exhibit strong antifungal activity in vitro may perform differently in fresh pasta systems, either losing efficacy or, conversely, displaying enhanced activity [98].

The combined antifungal effect of LAB cannot be explained by individual metabolites but rather by strain-specific, matrix-dependent synergetic effects. Investigations on individual compounds often fail to capture this multifaceted nature, which may partly explain the discrepancies in efficacy reported across different food systems. This points to the necessity of metabolite-solved but matrix-confirmed methods in fresh pasta studies. Therefore, the selection of bioprotective LAB strains must account not only for their metabolite profiles but also for their functional performance within the actual pasta matrix under realistic processing and storage conditions.

## 5. Application and Efficacy in Pasta Systems

The ultimate challenge for LAB-based antifungal biopreservation strategies in fresh pasta systems is the successful translation of laboratory-scale findings into practical, real-world applications. This transition requires careful consideration of integration strategies, rigorous evaluation of antifungal efficacy under realistic processing and storage conditions, and a comprehensive assessment of the impact on overall product quality [99].

The implementation of bioprotective cultures in fresh pasta production involves complex interactions among microbial physiology, processing parameters, and intrinsic product characteristics. Understanding these dynamics is essential for the development of robust application strategies capable of delivering consistent and reproducible antifungal performance in commercial production environments.

### 5.1. The Incorporation of LAB into the Pasta-Making Process

The incorporation of LAB-based antifungal strategies into fresh pasta profoundly affects microbial survival, spatial distribution, and overall bioprotective performance. Multiple implementation approaches have been proposed, each with distinct advantages and limitations (Table 3). The simplest approach involves the direct addition of viable LAB during dough mixing, using frozen or freeze-dried cultures incorporated into the mixing water or flour [100]. This method is operationally straightforward and generally ensures homogeneous distribution; however, precise control of inoculation levels (typically 10^6^–10^7^ CFU g^−1^) can be challenging, and LAB viability may be compromised by subsequent processing steps, particularly thermal treatments.

Enhanced control of antifungal efficacy may be achieved through the use of pre-fermented ingredients. Specifically, a fraction of the flour is subjected to fermentation with selected LAB strains for 16–24 h prior to dough preparation, generating a metabolite-rich fermentate that is subsequently incorporated into the formulation in liquid or dried form [101]. This approach ensures early availability of bioactive compounds, promotes strain adaptation to the cereal matrix, and allows potential sensory issues to be identified and addressed at an early production stage. However, it requires dedicated fermentation facilities and careful process control, which may increase operational complexity [11,102].

An alternative strategy involves the use of cell-free supernatants (CFSs), which contain concentrated antifungal metabolites without viable LAB cells. Incorporation of CFS into the dough circumvents challenges related to microbial survival and ensures antifungal activity independent of cell viability in the final product. Nevertheless, CFS formulations can be difficult to standardize, may adversely affect sensory attributes, and do not provide continued metabolite production during storage [103].

Surface application methods, such as spraying or dipping extruded pasta with concentrated LAB cultures or metabolite preparations, target the primary site of fungal contamination and are particularly effective when combined with packaging systems that maintain surface moisture [97]. However, this approach offers limited internal protection and typically requires additional processing equipment.

Processing parameters strongly influence LAB survival and performance. Mixing time and intensity affect culture dispersion and oxygen exposure, extrusion pressure and temperature impact cell viability, and drying or cooling conditions influence both LAB survival and product quality [104]. Scaling up from laboratory to industrial production introduces further variables, including differences in mixing efficiency, temperature control, and production scheduling, which may not directly translate from lab-scale conditions.

Therefore, successful implementation of antifungal LAB in fresh pasta production requires alignment of the chosen integration strategy with facility capabilities, shelf-life objectives, regulatory constraints, and the specific physiological characteristics of the selected LAB strain.

According to the literature reviewed, the evidence of LAB biopreservation of fresh pasta can be placed into three levels, i.e., (i) indirect evidence in the form of cereal or sourdough systems, (ii) model-matrix studies that mimic pasta composition, and (iii) actual in situ fresh pasta studies. Most of the present information is currently at the levels (i) and (ii), indicating low direct validation of real fresh pasta systems.

### 5.2. The Assessment of Shelf-Life Extension and Fungal Inhibition

The evaluation of antifungal LAB in fresh pasta must be conducted under conditions that closely replicate real storage and distribution environments. The cornerstone of this evaluation is the challenge test, in which pasta treated with selected LAB strains is deliberately inoculated with representative spoilage fungi, typically *Penicillium* or *Aspergillus* species, at concentrations of approximately 10^2^–10^4^ spores g^−1^ or via controlled surface contamination. Samples are then stored under defined humidity conditions at refrigeration temperatures (4–8 °C) to simulate commercial distribution [105].

Antifungal efficacy is commonly assessed by monitoring visible mold development, which provides a direct and practical indicator of shelf-life extension [106]. More quantitative approaches include longitudinal fungal enumeration to generate growth curves, as well as digital image analysis to objectively quantify mycelial coverage over time. The use of multiple complementary metrics enables a more robust characterization of inhibition dynamics and treatment effectiveness [107].

Importantly, mycotoxin analysis is a critical component of validation, as delayed fungal growth does not necessarily equate to suppressed toxin production. Key mycotoxins, such as ochratoxin A and aflatoxins, should therefore be quantified using validated analytical techniques, including High-Performance Liquid Chromatography–Tandem Mass Spectrometry (HPLC–MS/MS) or Enzyme-Linked Immunosorbent Assay (ELISA) assays [108]. Notably, several LAB strains have been shown to attenuate mycotoxin biosynthesis even when fungal growth is not completely inhibited, likely through interference with regulatory pathways involved in secondary metabolism. This effect represents an additional food safety benefit that may not be evident from growth-based assessments alone.

Environmental challenge tests, in which LAB-treated pasta is exposed to the naturally occurring fungal community within production facilities, further enhance the ecological relevance of validation studies. Although these tests better reflect real-world microbial complexity and can reveal protection against unforeseen contaminants, they are inherently less controlled and therefore require careful experimental design to ensure reproducibility and meaningful interpretation of results.

Mechanistic insight can be gained by monitoring the production and stability of antifungal metabolites, such as organic acids and PLA, throughout storage using analytical techniques such as HPLC or LC–MS. Tracking metabolite dynamics over time allows identification of the most effective strains, optimization of inoculation levels and application strategies, and evaluation of whether antifungal compounds are produced primarily during processing or continue to accumulate during storage [109].

Shelf-life extension is commonly evaluated using a combination of accelerated storage tests conducted at elevated temperatures and real-time storage studies. Although accelerated tests offer rapid preliminary insights, real-time evaluations remain essential for final validation, as fungal growth responses to temperature are non-linear and cannot be reliably predicted by extrapolation [110]. Comprehensive validation protocols should also incorporate temperature-abuse scenarios reflecting potential deviations during distribution and retail handling. Shelf-life extension that is reported to be achieved using antifungal lactic acid bacteria greatly relies on storage conditions and experimental design [40]. Most of the studies that have reported a delay in fungal growth were conducted under refrigerated storage (usually 4–8 °C) or controlled ambience conditions and with specified packaging systems and inoculated spoilage fungi [43,44]. The differences in LAB strain, food matrix, application mode, and target microorganism help significantly in achieving the desired preservation effects.

To enhance transparency and to aid the reported preservation effects, key research that analyzes antifungal LAB in fresh pasta and other associated cereal-based products is presented in Table 4. It is important to mention that direct in situ research on antifungal preservation with the use of LAB in fresh pasta is still scarce in comparison to other cereal types, hence the necessity of specific validation in pasta-specific processing and storage circumstances. The biggest drawback of the reviewed studies is that too much reliance was present on agar-based inhibition assays, which often exaggerated the antifungal effect in comparison to actual pasta systems. Very few studies used in situ challenge tests with fresh pasta matrices, and even fewer investigated the production of mycotoxins in association with fungal growth. Consequently, antifungal effects, which are reported, cannot in every case be extrapolated to commercial fresh pasta conditions.

### 5.3. Impact on Pasta Quality and Sensory Properties

Beyond antifungal efficacy, the effective use of bioprotective LAB in fresh pasta requires the maintenance of product quality over the entire shelf life.

One of the primary risks associated with LAB application concerns visual appearance. Certain LAB strains may induce slight darkening or off-yellow coloration, potentially linked to pH-dependent reactions or pigment-associated metabolic activity [111]. These effects are highly strain-specific and can generally be mitigated through careful strain selection and optimization of inoculation levels.

Textural properties constitute another sensitive quality parameter in fresh pasta. LAB-induced acidification may affect gluten network formation, while enzymatic activities can weaken starch or protein structures, and gas production may introduce undesirable porosity. These changes are commonly evaluated through instrumental texture analyses, including measurements of firmness, elasticity, stickiness, and cooking loss. When appropriately selected and applied at controlled dosages, most LAB strains exert minimal effects on texture; however, highly proteolytic or amylolytic strains, or extended storage periods, may result in softening or increased stickiness [112].

Flavor and aroma are often the most sensitive quality attributes affected by LAB metabolism. LAB can produce a range of volatile compounds that impart sour, bitter, or cheese-like notes, although some strains are largely sensorially neutral or may even enhance flavor complexity [113]. Because these sensory changes evolve over storage, evaluation by trained sensory panels and consumer acceptance testing is essential.

Additional quality aspects also warrant consideration, including lipid stability in egg-enriched pasta, pH-related effects on cooking behavior and starch digestibility, and interactions between LAB-derived exopolysaccharides and water retention within the pasta matrix [114]. Collectively, these factors underscore the need for comprehensive, multi-parameter quality assessment when implementing bioprotective LAB.

Several strategies may be employed to mitigate potential quality impacts while maintaining antifungal efficacy. The use of mixed LAB cultures can help balance antimicrobial activity and sensory outcomes; adjustments in fermentation conditions may modulate metabolite profiles; and encapsulation techniques can enable controlled release of bioactive compounds. Furthermore, natural flavor synergies can be leveraged by matching specific LAB strains to pasta formulations, such as whole-wheat or herb-enriched products [100]. Advances in predictive modeling of metabolite profiles and their sensory outcomes may further facilitate targeted strain selection. Ultimately, the successful application of antifungal LAB in fresh pasta depends on achieving a careful balance between microbial protection and sensory integrity, ensuring both extended shelf life and consumer acceptance.

## 6. Industrial Perspectives

The translation of laboratory-scale success to industrial fresh pasta manufacturing remains a complex and multifactorial challenge. Compared with industrial fermentation systems (1000–10,000 L), bench-scale fermentations (1–10 L) differ substantially in terms of oxygen transfer rates, heat dissipation, shear stress, nutrient gradients, and, consequently, biomass formation and metabolite profiles. In parallel, variability in raw materials, such as flour type, indigenous microbial load, amino acid composition, and egg ingredients, can markedly influence the performance and consistency of antifungal LAB cultures [115]. Additional stresses are imposed during industrial mixing, extrusion, and thermal processing, while the resident microbiota and environmental fungal populations encountered in manufacturing facilities often differ significantly from laboratory model organisms [116]. Addressing these discrepancies requires the selection of stress-tolerant strains, the application of physiological preconditioning, optimization of inoculation levels, and targeted adaptation of processing parameters.

From a regulatory perspective, antifungal LAB intended for commercial use must comply with food safety frameworks such as Generally Recognized as Safe (GRAS) status in the United States or Qualified Presumption of Safety (QPS) status in the European Union [117]. Accurate taxonomic identification through genomic and phenotypic profiling is essential to exclude pathogenic or opportunistic relatives. Whole-genome sequencing has become a key tool to verify the absence of virulence factors, biogenic amine biosynthesis pathways, and transferable antibiotic resistance genes [118]. While strains with a documented history of safe use offer clear regulatory advantages, novel isolates typically require extensive in vitro and in vivo safety evaluation. Moreover, because bioprotective LAB may persist throughout a product’s shelf life, consumer exposure levels can exceed those associated with conventional starter cultures, necessitating comprehensive risk assessment, particularly for vulnerable consumer groups. Early engagement with regulatory authorities is therefore critical, as approval timelines may extend over several months or even years [119].

Economic feasibility represents a further determinant of successful commercialization. Large-scale fermentation, downstream processing, and stabilization technologies such as freeze-drying or spray-drying entail significant costs, although they enhance culture stability, extend shelf life, and may reduce required inoculation levels [119]. Commercial formulations must ensure adequate culture viability and metabolite production over 12–24 months, while balancing cost efficiency, labeling requirements, and storage constraints. The economic benefits associated with shelf-life extension, including reduced food waste, improved logistical flexibility, and access to premium market segments, must clearly outweigh these additional costs. Consumer acceptance is strongly influenced by clean-label positioning and transparent, non-technical communication, while brand strategy, retailer requirements, competition with alternative preservation technologies, and regional regulatory differences further shape market adoption [120].

In addition to the well-documented technological and safety benefits, the implementation of antifungal LAB can be explicitly linked to tangible economic advantages for manufacturers. By significantly extending the shelf life of fresh pasta, LAB-based biopreservation directly reduces product spoilage, returns, and recalls associated with fungal growth, thereby lowering food waste along the supply chain, from production and distribution to retail [121]. This reduction in waste translates into measurable cost savings that can partially or fully offset the higher initial costs associated with LAB culture production, formulation, and process adaptation.

Moreover, LAB-enabled formulations align strongly with the clean-label positioning of fresh pasta, which is increasingly perceived by consumers as a premium product category. Clean-label claims based on natural biopreservation allow manufacturers to justify premium pricing strategies, as consumers are demonstrably willing to pay more for products perceived as safer, more natural, and more sustainable [122]. In this context, LAB should be viewed not merely as a cost factor but as a value-generating investment that simultaneously enhances shelf life, reduces economic losses due to spoilage, supports sustainability goals through waste reduction, and strengthens brand differentiation in the premium fresh pasta market. Ultimately, the broader benefits of antifungal LAB, including extended shelf life, reduced product losses, and clean-label product development, can only be achieved through the simultaneous consideration of industrial-scale performance, regulatory compliance, and economic viability [123]. The successful translation of laboratory efficacy into commercial reality therefore depends on integrated strain selection, formulation design, and process optimization within an industrially relevant framework.

Although several laboratory- and pilot-scale studies have demonstrated the antifungal potential of LAB in cereal-based foods, fully documented industrial case studies on the application of LAB in fresh pasta processing remain scarce. Key challenges include the successful introduction of selected strains into large-scale fermentations, strain stability, consistent metabolite production, integration into existing processing lines, and the maintenance of sensory quality during extended storage. In addition, regulatory acceptance, process repeatability, and cost–benefit considerations represent major barriers to industrial adoption. Collectively, these limitations indicate that, while LAB-based biopreservation of fresh pasta is technologically promising, it still requires systematic validation at the industrial scale.

## 7. Future Implications

Future advancements in the application of antifungal lactic acid bacteria (LAB) will largely depend on two converging pillars: smarter strain discovery and targeted process optimization. The increasing availability of genomic tools and genome mining approaches enables early prediction of antifungal potential, thereby reducing reliance on costly and time-consuming empirical screening at advanced laboratory stages [124]. While metabolic pathway engineering could, in principle, be used to enhance the production of key antifungal metabolites and suppress undesirable by-products, regulatory constraints and consumer resistance toward genetically modified organisms (GMOs) remain significant barriers to widespread adoption.

The integration of multi-omics approaches, including transcriptomics, proteomics, and metabolomics, offers unprecedented insight into LAB physiology and metabolite production under food-relevant conditions. Such datasets can support rational process design and facilitate a deeper understanding of strain performance within complex food matrices [125]. As a result, strain selection is expected to become increasingly predictive rather than empirical, supported by automated high-throughput screening platforms operated under matrix-mimicking conditions that more accurately reflect industrial environments [126].

Importantly, future preservation strategies are unlikely to rely on LAB alone. Instead, LAB-based biopreservation is expected to be integrated into multi-hurdle systems. Synergistic combinations with natural antimicrobials, such as plant extracts or essential oils, may enhance antifungal efficacy while maintaining clean-label compliance [127].

In addition, mild physical treatments, including heat, high-pressure processing (HPP), or pulsed electric fields (PEFs), can be applied to reduce initial microbial loads without compromising LAB viability. Although there are no studies evaluating the combined effect of non-thermal preservation technologies and LAB by studying the combined effects on different food matrices, we can hypothesize that the incorporation of protective LAB cultures into pasta dough or fillings enhances the effectiveness of HPP. In fact, LAB strains such as *Lactiplantibacillus plantarum*, *Pediococcus acidilactici*, and bacteriocin-producing *Lactococcus lactis* can survive moderate-pressure treatments and inhibit the recovery of pressure-injured cells during refrigerated storage [128,129,130]. This synergy allows for reduced processing intensity while maintaining microbiological safety.

In filled pasta products (e.g., ravioli or tortellini), LAB can be selectively added to dairy- or meat-based fillings, which are particularly susceptible to post-processing contamination. Studies have shown that the combination of LAB and HPP effectively controls *Listeria monocytogenes*, extending shelf life from approximately 10–14 days to up to 60 days under refrigerated conditions [131].

However, careful strain selection is required to avoid excessive acidification, which may negatively impact sensory properties.

PEF technology is mainly applicable to liquid or semi-liquid matrices; consequently, its direct use in solid foods such as pasta is limited. However, indirect PEF applications may enhance LAB functionality during pasta production. PEF can be used to treat liquid ingredients such as egg products, water, or milk prior to dough preparation, reducing microbial load while preserving functional properties. Mild PEF treatments may also selectively reduce competing microflora in pasta fillings, allowing LAB to dominate during storage. Additionally, sublethal PEF exposure has been reported to enhance membrane permeability and metabolic activity in LAB, potentially accelerating fermentation-related processes or improving stress tolerance [132,133]. Despite these promising effects, industrial-scale application of PEF in pasta processing remains under development.

Future research should focus on the selection and encapsulation of pressure-tolerant LAB strains, the optimization of HPP parameters to achieve an optimal balance between microbial inactivation and LAB survival, the development of symbiotic fresh pasta products enriched with prebiotic fibers, and the validation of combined non-thermal technologies at the industrial scale. Overall, the integration of LAB with non-thermal processing technologies represents a sustainable and effective strategy to enhance the safety and shelf life of fresh pasta while preserving its nutritional, sensory, and technological quality.

In parallel, modified atmosphere packaging (MAP) may help stabilize the environmental conditions required for sustained LAB activity. The development of designed microbial consortia further offers the potential to broaden antifungal coverage and extend protection over time. Marzano et al. [134] evaluated the effects of modified atmosphere composition and packaging, with or without the incorporation of bioprotective cultures (*Lactobacillus acidophilus*, *Lactobacillus casei*, *Bifidobacterium* spp., and *Bacillus coagulans*) into semolina, on the microbial quality, safety, and shelf life of fresh pasta. Despite limited growth of some bioprotective cultures, spoilage and food-borne microbiota were effectively controlled in fresh pasta through synergistic antimicrobial and fermentation metabolites. Consequently, the combined application of MAP and bioprotective cultures reduced quality loss and extended the refrigerated shelf life of fresh pasta by up to 30 days.

Emerging intelligent packaging solutions, capable of controlling oxygen and moisture levels while simultaneously monitoring system performance, represent an additional layer of control [135].

Although this review focuses on fresh pasta, these concepts are broadly applicable to other high-moisture cereal-based foods, including fresh noodles, par-baked doughs, gluten-free products, plant-based matrices, and traditional cereal foods. However, successful implementation in each case requires product-specific strain selection and precise optimization of processing conditions [11,18].

In summary, antifungal LAB should be viewed not as a stand-alone solution but rather as a sustainability-oriented tool capable of reducing chemical preservative use, minimizing food waste, and preserving product quality. Their effectiveness depends on integration within a comprehensive quality management framework that addresses raw material variability, hygiene practices, and processing conditions [136].

The prevailing trend in food biopreservation is toward precision—aligning specific LAB strains with defined product characteristics and processing conditions—rather than relying on generic culture applications. Achieving this objective requires close integration of microbiology, process engineering, and industrial realities to ensure both technological feasibility and commercial success [137].

## 8. Conclusions

The growing demand for clean-label foods has intensified interest in natural preservation strategies, positioning antifungal lactic acid bacteria (LAB) as a viable and sustainable alternative for controlling fungal spoilage in fresh pasta, a high-moisture product particularly susceptible to contamination by molds such as *Penicillium* and *Aspergillus*. LAB strains are isolated from cereal-based ecosystems, most notably *Lactiplantibacillus plantarum*, showing strong adaptation to grain-derived matrices and the ability to produce a broad spectrum of antifungal metabolites. This synergistic metabolite repertoire, including organic acids, phenyllactic acid, and cyclic dipeptides, acts through multiple complementary mechanisms such as cytoplasmic acidification, disruption of fungal energy metabolism, and damage to cell membrane integrity, and in several cases is also associated with a reduction in mycotoxin biosynthesis, thereby enhancing food safety beyond spoilage control alone. However, the successful implementation of LAB-mediated biopreservation requires careful strain selection that balances antifungal efficacy with technological suitability, sensory neutrality, and compliance with established regulatory frameworks such as GRAS and QPS, as well as thoughtful integration into fresh pasta production through direct inoculation, the use of pre-fermented ingredients, or surface application, all while accounting for industrial scale-up challenges including raw material variability, processing constraints, and storage conditions. Although the benefits of shelf-life extension, food waste reduction, and alignment with consumer clean-label expectations provide strong incentives for industrial adoption, the application of LAB antifungal biopreservation in fresh pasta remains limited at the industrial level. This limitation is primarily related not to a lack of efficacy but rather to the difficulty of ensuring reproducible performance under commercial processing and storage conditions. According to the literature reviewed, the evidence supporting LAB-based biopreservation of fresh pasta can be classified into three levels, namely indirect evidence from cereal or sourdough systems, studies conducted in model matrices that mimic pasta composition, and true in situ investigations performed directly on fresh pasta, with most of the available data currently concentrated in the first two levels, indicating a clear gap in direct validation under real fresh pasta conditions. Until standardized protocols for in situ challenge testing under industrially relevant conditions are developed, LAB-mediated biopreservation of fresh pasta should therefore be considered a promising but not yet fully mature technology. Overall, existing evidence supports the potential of LAB-based antifungal strategies for fresh pasta preservation, but their application is still based on limited pasta-specific data and requires confirmation at the industrial scale. Future developments are expected to rely on genomics-driven strain selection, the integration of LAB with other natural antimicrobials or mild physical preservation treatments, and the extension of these strategies to a wider range of high-moisture cereal-based foods, with continued interdisciplinary collaboration among microbiologists, food technologists, process engineers, and industry stakeholders being essential to fully realize the commercial potential of this approach.

## Figures and Tables

**Table 3:** Integration strategies for antifungal LAB in fresh pasta production.

Integration Strategy	Description	Main Advantages	Limitations/Challenges	Technological and Industrial Implications	Sensory Impact	TRL Level	Industrial Readiness	Ref.
Direct addition of viable LAB	Inoculation of frozen or freeze-dried LAB during dough mixing via water or flour	Simple implementation; homogeneous distribution; low initial costs	Limited control of inoculum level (10^6^–10^7^ CFU g^−1^); reduced viability during thermal/mechanical steps	Strong dependence on processing parameters; efficacy linked to cell survival during shelf life	Low–moderate (strain- and dose-dependent)	TRL 7–8	High (minimal process modification)	[100]
Pre-fermented ingredients	Pre-fermentation (16–24 h) of a flour fraction with selected LAB, added as a liquid or dried fermentate	Early availability of metabolites; better strain adaptation; early sensory control	Requires dedicated fermentation facilities; higher process complexity	Improved reproducibility; increased operational costs	Moderate (can be controlled during fermentation)	TRL 6–7	Medium–high (requires additional unit operation)	[11,101,102]
Cell-free supernatants (CFSs)	Addition of concentrated antifungal metabolites without live cells	Activity independent of cell viability; immediate antifungal effect	Difficult standardization; possible sensory impact; no metabolite renewal	Precise dosage control required; limited long-term protection	Moderate–high (risk of off-flavors)	TRL 5–6	Medium (regulatory and sensory constraints)	[103]
Surface application (spraying/dipping)	Treatment of extruded pasta surface with LAB or metabolites	Targeted action at contamination sites; effective with moist packaging	Limited internal protection; additional equipment required	Highly effective with MAP; flexible post-extrusion application	Low (localized effect)	TRL 6–7	Medium (equipment and line adaptation needed)	[97]
Process parameter optimization	Control of mixing, extrusion, and cooling/drying conditions	Improved LAB survival and distribution	High variability during industrial scale-up	Requires plant-specific optimization; critical for reproducibility	Indirect (process-mediated)	TRL 7–9	High (integrated into existing QA systems)	[104]

**Table 4:** Shelf-life extension by antifungal LAB.

Food Matrix	LAB Strain(s)/Treatment	Target Fungus	Storage Conditions	Shelf-Life Extension (%)/Outcome	Reference
Fresh semolina pasta fortified with chickpea sourdough fermented with selected LAB	*Lactiplantibacillus plantarum* + *Furfurilactobacillus rossiae* starter (chickpea sourdough)	*Penicillium roqueforti* DPPMAF1 (artificially inoculated)	4 °C, sealed bags, 40-day storage	Longer mold-free period vs. control/calcium propionate; >40 days before visible colonies < control (<40 days)	[11]
Sourdough bread	*Lactobacillus* spp. sourdough fermentate	Various molds	Ambient (bread storage)	~10–28 days of extended mold-free shelf life vs. control (varies by strain/product)	[46]
Sourdough bread	Selected sourdough *Lactobacilli* with antifungal activity	Bread spoilage fungi	Ambient (bread storage)	~7–14-day extension vs. control (varies by strain/product)	[55]

## Data Availability

All information produced or examined in this research is incorporated within this published article.
